# The GPVI-Fc Fusion Protein Revacept Improves Cerebral Infarct Volume and Functional Outcome in Stroke

**DOI:** 10.1371/journal.pone.0066960

**Published:** 2013-07-23

**Authors:** Silvia Goebel, Zhongmin Li, Jasmin Vogelmann, Hans-Peter Holthoff, Heidrun Degen, Dirk M. Hermann, Meinrad Gawaz, Martin Ungerer, Götz Münch

**Affiliations:** 1 AdvanceCOR GmbH (formerly Procorde GmbH), Martinsried, Germany; 2 Department of Neurology, Universitätsklinikum Essen (D.M. H.), Essen, Germany; 3 Department of Internal Medicine III, Universität Tübingen (M.G.), Tübingen, Germany; Julius-Maximilians-Universität Würzburg, Germany

## Abstract

**Objectives:**

We examined the effect of Revacept, an Fc fusion protein which is specifically linked to the extracellular domain of glycoprotein VI (GPVI), on thrombus formation after vessel wall injury and on experimental stroke in mice.

**Background:**

Several antiplatelet drugs for the treatment of myocardial infarction or ischemic stroke with potent anti-ischemic effects have been developed, but all incur a significant risk of bleeding.

**Methods:**

Platelet adhesion and thrombus formation after endothelial injury was monitored in the carotid artery by intra-vital fluorescence microscopy. The morphological and clinical consequences of stroke were investigated in a mouse model with a one hour-occlusion of the middle cerebral artery.

**Results:**

Thrombus formation was significantly decreased after endothelial injury by 1 mg/kg Revacept IV, compared to Fc only. 1 mg/kg Revacept IV applied in mice with ischemic stroke immediately before reperfusion significantly improved functional outcome, cerebral infarct size and edema compared to Fc only. Also treatment with 10 mg/kg rtPA was effective, and functional outcome was similar in both treatment groups. The combination of Revacept with rtPA leads to increased reperfusion compared to treatment with either agent alone. In contrast to rtPA, however, there were no signs of increased intracranial bleeding with Revacept. Both rtPA and Revacept improved survival after stroke compared to placebo treatment. Revacept and vWF bind to collagen and Revacept competitively prevented the binding of vWF to collagen.

**Conclusions:**

Revacept reduces arterial thrombus formation, reduces cerebral infarct size and edema after ischemic stroke, improves functional and prognostic outcome without intracranial bleeding. Revacept not only prevents GPVI-mediated, but probably also vWF-mediated platelet adhesion and aggregate formation. Therefore Revacept might be a potent and safe tool to treat ischemic complications of stroke.

## Introduction

Ischemic stroke is the most frequent disabling disease and a leading cause of death above the age of 60 years [1]. Most frequently, the underlying cause is rupture of atherosclerotic plaques which leads to platelet adhesion and thrombus formation or embolisation in cerebral arteries [2]. GPVI-mediated and von Willebrand Factor (vWF)-mediated platelet adhesion and activation play an important role in thrombus formation and subsequent development of stroke and could be a target for pharmacological inhibition of pathological thrombus formation [3].

vWF binds to its platelet receptor GPIb and plays an important role in primary hemostasis (see elsewhere for a review [4], [5]). GPVI is the major signalling receptor for collagen and exclusively expressed on platelets and megakaryocytes initiating platelet recruitment at sites of vascular injury [6], [7]. Both blocking of GPIbα and GPVI with specific antibodies led to a reduced infarct volume and a significantly improved functional outcome in an acute stroke model in mice with one hour occlusion of the middle cerebral artery (MCA) [8]. This finding was confirmed in vWF-/- mice [9]. These animals did not show any increased incidence of intracranial haemorrhage but tail bleeding time was increased in mice treated with anti-GPIbα antibodies.

Despite tremendous progress in the understanding of the mechanisms of plaque-induced thrombus formation and development of novel anti-platelet drugs, the progress did mostly not translate into improvement of patients care with TIA or stroke: recently a clinical phase III trial (AbESTT-II) with a novel anti-platelet drug was discontinued due to increased fatal intracranial haemorrhage and poor outcomes [10].

Interestingly, recent clinical studies underlined the importance of GPVI-mediated signalling. Increased GPVI mediated platelet activation and a subsequent shedding of GPVI was determined in the blood of patients with acute vascular syndromes [11], [12]. Inhibition of GPVI-mediated platelet activation can be achieved both by anti-GPVI antibodies and by the soluble GPVI receptor. Revacept, a dimeric soluble GPVI-Fc fusion protein, was recently tested in a clinical phase I study. It was shown to be a safe and well-tolerated new anti-platelet compound with a clear dose-dependent pharmacokinetic profile. Revacept led to an inhibition of platelet aggregation but unaltered general hemostasis in all subjects [13].

In contrast to other anti-platelet approaches, soluble GPVI-Fc binds to atherosclerotic endothelium both with and without plaque rupture [14]. This lesion-directed approach should have valuable advantages with high spatial selectivity at the site of plaque-induced thrombus formation. Moreover, as Revacept addresses vascular collagen, it might also interfere with other collagen-dependent pathways including alpha_2_/beta_1_ integrins or vWF-mediated GPIb activation.

Based on this hypothesis, we tested Revacept for inhibition of thrombus formation, cerebral damage and pre-clinical outcome after experimental arterial thrombosis in different models including a stroke animal model. We compared the effects to the only established pharmacological intervention in stroke for patients, recombinant tissue plasminogen activator (rtPA).

## Methods

### Experimental groups and materials used

As therapeutic tool we used Revacept, a dimeric soluble GPVI-Fc protein which was produced as previously described [13] as well as the Fc part from human IgG for the control group [15]. In various interventions, recombinant tissue plasminogen activator (rtPA, Actilyse, Boehringer Mannheim, Germany) was used.

### ELISA to determine binding of Revacept or vWF to collagen

An ELISA was established to evaluate binding of Revacept or human vWF to mouse or bovine collagen I (for details see Methods S1). Competition of human vWF factor to collagen by Revacept was also determined.

### Animal studies for the Determination of platelet function *in vivo* and morphological and functional outcome after ischemic stroke

Experiments were approved by the local animal welfare authority in Munich, Germany (Regierung von Oberbayern, Sachgebiet Tierschutz, reference number 55.2-1-54-2531-98-09). To test the antithrombotic effect of Revacept on an injured arterial wall, a lesion of the endothelium was induced by transient ligature of the left common carotid artery (ACC) as previously described [7]. To visualize platelet adhesion to the injured vessel wall under in vivo conditions, intravital microscopy was performed after administration of Revacept or Fc only, immediately before inducing the endothelial lesion in the ACC (for details see Methods S1).

The influence of Revacept on arterial thrombosis induced by deeper lesions of the arterial wall was investigated in a mouse model of wire-induced vascular injury. Thrombus size was quantified after digital imaging and quantification by image analysis software (Photoshop, Adobe).

The effect of Revacept on cerebral infarction and neurological function/motor activity after cerebral ischemia was assessed in mice after occlusion of the left middle cerebral artery (MCA). At the beginning of reperfusion, Revacept or Fc only, respectively, were injected via the tail vein. Revacept has a terminal half life in mice of approximately 12 hours. rtPA (10 mg/kg body weight) was also studied in this stroke model, as well as the combination of rtPA with Revacept. After reperfusion times of 4 hours, 24 hours and 72 hours, mice underwent evaluation for neurological and motor dysfunction. Survival after stroke was monitored 4, 24 and 72 hours after reperfusion (see details in Methods S1). In post mortem analyses, histological evaluation of infarct area with 2,3,5-triphenyltetrazolium chloride (TTC; Sigma Aldrich No 93140) and for the evaluation of the edema area was assessed. Brain slices were digitally photographed and the infarct size was quantified by image analysis software (Photoshop® CS5, Adobe) by researchers blinded to the treatment groups. The hemoglobin content of brains was quantified with a spectrophotometric assay (for further details please see Methods S1).

### Immunstaining for IgG, Revacept, macrophages, TGF-ß, and PDGF

Immunstaining was done with tissue sections obtained from mouse brains (for further details please see Methods S1).

### Statistical methods

Results are presented as mean ± standard error of the mean (SEM). Statistical analysis was performed using an independent T-test for pairwise comparison, and one-way ANOVA followed by a post hoc analysis (Fisher Least significant Difference tests for multiple comparisons) for multiple comparisons, where appropriate. Adjustment for multiple testing of time was made for comparison of Doppler flow measurements. The Logrank test was applied for the analysis of the differences in the mortality. Values of p<0.05 were considered as statistically significant.

## Results

### Binding of Revacept and vWF to collagen and competition of binding to collagen by Revacept

**Figure 1:**
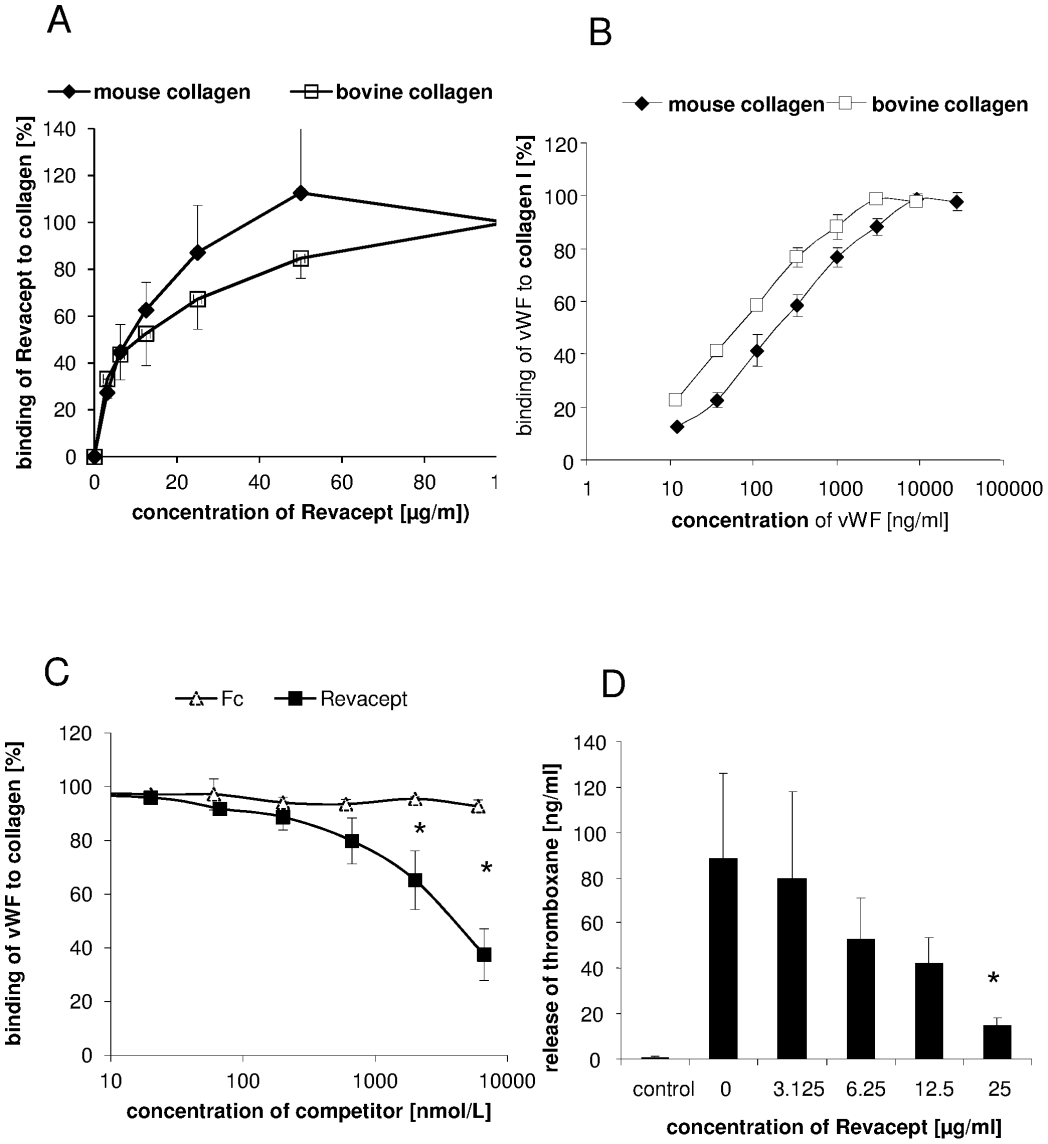
In vitro binding characteristics of Revacept. (A) Concentration-dependent binding of Revacept to immobilized mouse or bovine collagen on microtiter plates. (B) Increasing concentrations of von Willebrand factor (vWF) also bind to immobilized mouse or bovine collagen type I on microtiter plates. (C) Increasing amounts of Revacept lead to a competition with vWF for collagen binding. In contrast, Fc only did not show any inhibitory effect when used in equimolar amounts. Mean ± SEM are given, n = 4 per group, * represents statistical significance of p<0.05. (D) Effect of increasing concentrations of Revacept on release of thromboxane from human platelets. Mean ± SEM are given, n = 8 per group, * represents statistical significance of p<0.05 compared to Revacept 0 µg/ml.

Revacept showed specific and linear dose-dependent binding to both mouse and bovine collagen, as previously described (Figure 1A). Similarly, dose-dependent binding of vWF to mouse or bovine collagen type I coated on ELISA microtiter plates was demonstrated with half maximum bindings of 262 and 82 ng/ml, respectively (Figure 1B). In a competitive ELISA experiment, Revacept at increasing doses competed for the binding of vWF to collagen whereas Fc only did not exhibit this effect (see Figure 1C). Additionally, Revacept dose-dependently inhibited CRP-(collagen-related peptide)-stimulated thromboxane release from human platelets (Figure 1 D).

### Effect of Revacept on platelet-endothelial cell interactions and inhibition of thrombus formation after endothelial lesion in mice *in vivo*

**Figure 2:**
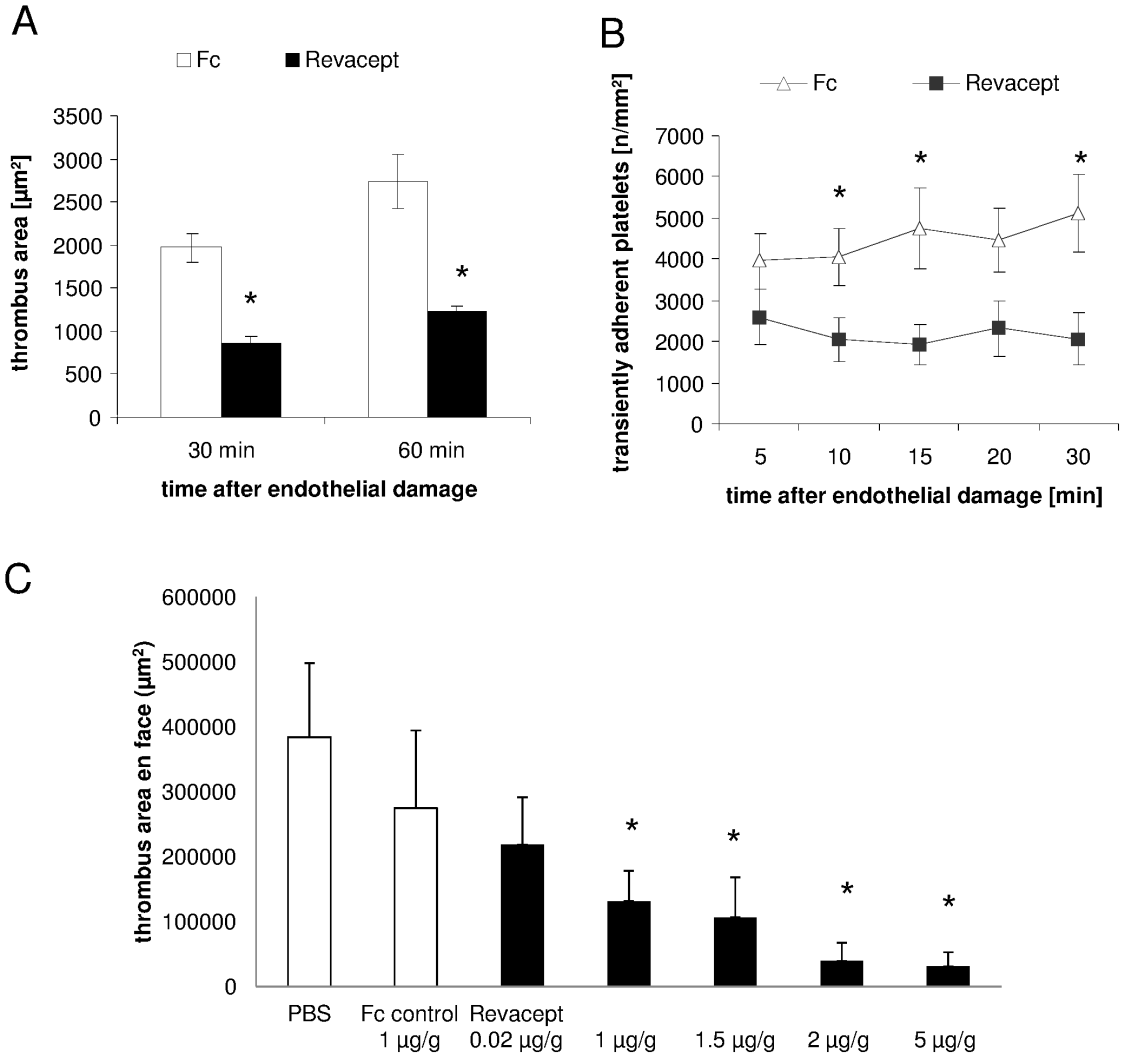
In vivo effects of Revacept on thrombus formation. Effect of Revacept (1 mg/kg body weight) on thrombus area 30 and 60 minutes after vascular injury induced in the left common carotid artery (A) and effect on transient platelet adhesion (B) of Revacept (1 mg/kg) compared to equimolar amount of Fc only (0.33 mg/kg). The agents were injected intravenously (tail vein) before experimental endothelial lesion. To visualize platelet adhesion and thrombus formation, mice received fluorescence-labelled platelets before injection of agents. Imaging of platelet aggregation was assessed in the carotid artery *in vivo* by using intravital microscopy (IVM). Mean ± SEM are given, n = 7 per group, * represents statistical significance of p<0.05. (C) Effect of Revacept (0.02–5 mg/kg body weight) on thrombus area after experimental deep vascular injury. The thrombus area was investigated post mortem in macroscopic en face preparations of the carotid area (in µm^2^). Increasing amounts significantly inhibit thrombus formation. (Mean ± SEM of n = 8–9 animals; * represents statistical significance of p<0.05 compared to PBS buffer).

In this model of transient ligature of the carotid artery, a superficial endothelial erosion could be verified by histological analysis, leading to vascular injury with consecutive thrombus formation. Representative images are shown in Figure S1. Revacept (1 mg/kg body weight) caused a significant reduction of platelet thrombus size 30 minutes as well as 60 minutes after endothelial damage in the left common carotid artery compared to the Fc only group (please see Figure 2A). The initial platelet adhesion to the ruptured endothelium, expressed as platelets per mm^2^ surface, was significantly reduced in the Revacept group at 10, 15 and 30 minutes after endothelial damage compared to the Fc only group (Figure 2B). Please find a representative in vivo fluorescence image of the carotid artery with endothelial lesion in mice in vivo in Figure S2.

### Effect of Revacept on thrombosis induced by deep vascular injury

Deep, wire-induced vascular injury leads to exposure of deep layers of the vascular wall to the blood. Revacept dose-dependently inhibited arterial thrombosis after wire-induced deep vascular lesion in mice. In this macroscopic post mortem analysis, doses of 1 µg/g and higher significantly inhibited arterial thrombosis compared to buffer control. Fc only used as control or lower doses of Revacept had no significant effect on arterial thrombus formation after experimental vascular lesion (see Figure 2C).

### Influence of Revacept on stroke in mice after middle cerebral artery (MCA) occlusion

**Figure 3:**
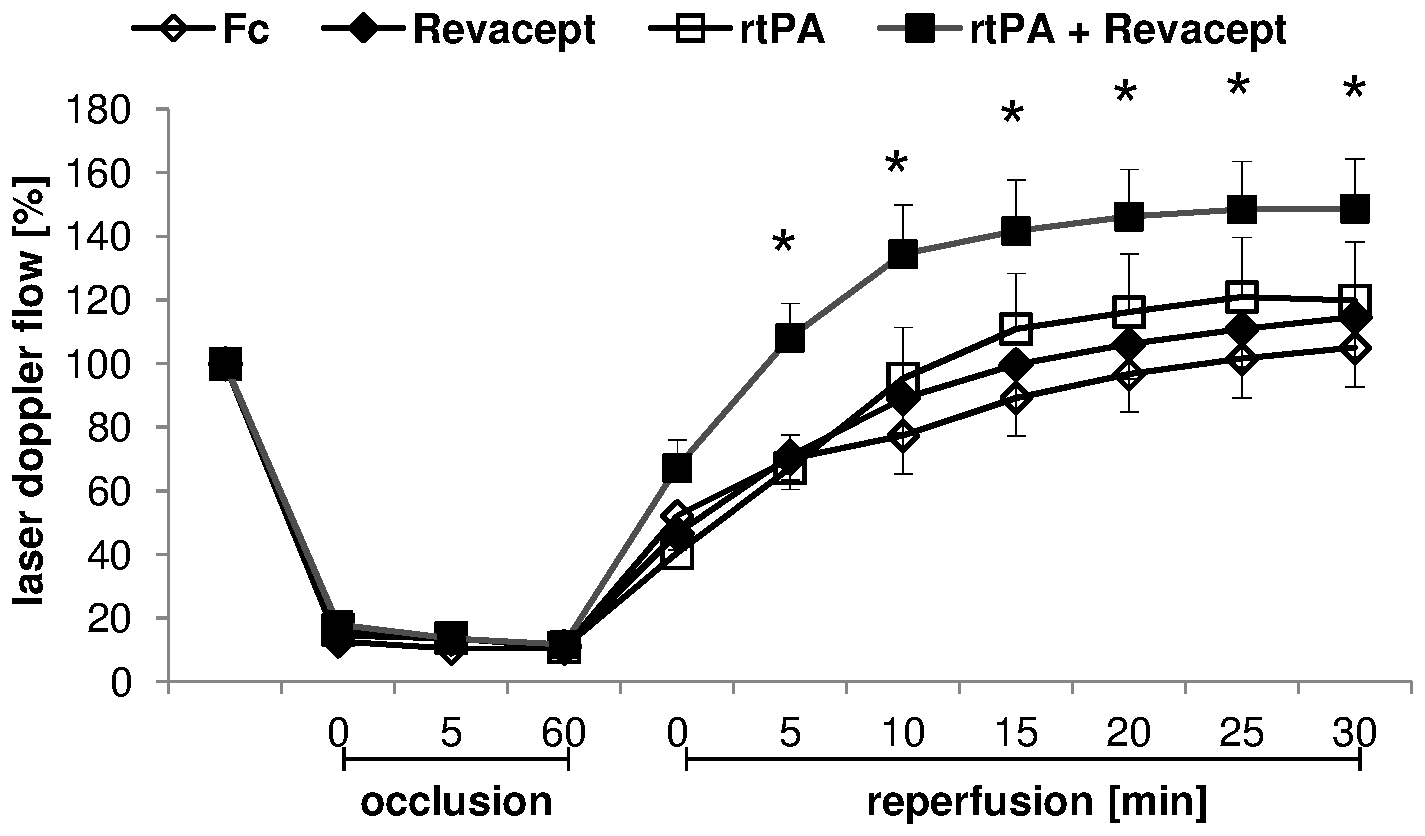
Experimental stroke model in mice. Flow in the middle cerebral artery (MCA) was measured after insertion of the catheter/filament and the necessary occlusion of the common carotid artery ( = 100%), during occlusion and 30 minutes of reperfusion, detected by a flexible Laser Doppler flow probe attached to the temporal skull. 1 mg/kg body weight Revacept, or the equimolar amount of Fc only (0.33 mg/kg), or rtPA (10 mg/kg), or a combination of Revacept and rtPA were injected intravenously during reperfusion of the MCA. Mean ± SEM are given, n = 8–10 per group. * represents statistical significance of p<0.05 compared to Fc.

Blood flow in the MCA was recorded during one hour occlusion and 30 minutes reperfusion. After MCA occlusion, the flow was constantly reduced by >80% during the occlusion time (see Figure 3). There were no differences between the groups during the occlusion time. With the onset of reperfusion, 10 mg/kg rtPA, 1 mg/kg Revacept, the combination of both or the equimolar amount of Fc only, respectively, were slowly injected into the tail vein. During reperfusion, no effects of the administrations of either Revacept alone or of rtPA alone were observed – both treatments resulted in comparable flow patterns. However, the combination of Revacept and rtPA resulted in relevantly better reperfusion compared to Fc control (Figure 3).

### Effect of Revacept on functional outcome and survival in mice after stroke induced by MCA occlusion

**Figure 4:**
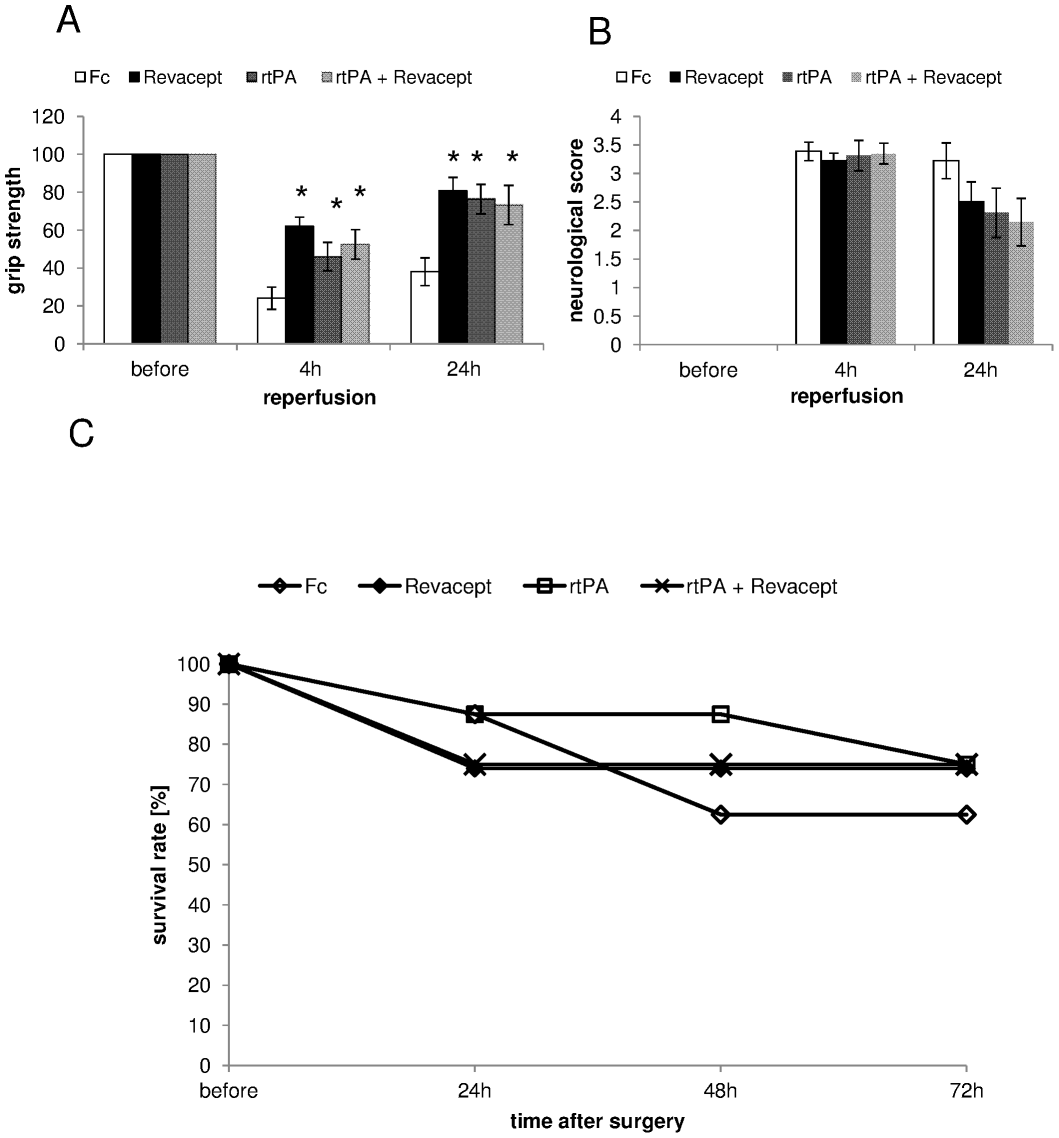
Assessment of functional outcome 4 hours and 24 hours after reperfusion following a 1 hour transient MCA occlusion. The grip strength (A) and the modified neurological Bederson score (B) (0 = no deficit, 1 = forelimb flexion, 2 = decreased resistance to lateral push without circling, 3 = circling, 4 = no spontaneous movement) was compared in mice treated with Revacept (1 mg/kg body weight), or the equimolar amount of Fc only (0.33 mg/kg), or rtPA (10 mg/kg), or a combination of Revacept and rtPA. The mean change in grip strength in percent compared to before surgery (100%) and the Bederson score is shown as mean ± SEM, n = 9 per group, * represents statistical significance of p<0.05 compared to Fc. (C) Survival rate after stroke in mice was assessed for up to 72 hours after stroke. rtPA; Actilyse 10 mg/kg, Revacept (1 mg/kg), or control were compared. N = 8 animals were investigated in the groups with this longer observation period.

Functional outcome was assessed 4 and 24 hours after the onset of reperfusion. Figure 4A shows a significant increase in grip strength with Revacept compared to Fc only at 4 as well as 24 hours after reperfusion. Both, rtPA and the combination of rtPA+Revacept showed significantly better outcomes but with a trend to less positive motor activity compared to Revacept. The neurological function is represented in Figure 4B. There were no statistically significant differences between the groups but we observed a tendency for better functional outcome in neurological function at 24 hours with either Revacept or rtPA alone. The combination therapy of both agents slightly missed statistical significance (p = 0.058 vs Fc, please see Figure 4B). Survival was assessed in mice four, 24 hours, 48 and 72 hours after reperfusion. The outcome of Revacept treated animals was compared to placebo treated animals with comparable injection volume. Moreover, the outcome after rtPA or both, Revacept and rtPA, was also assessed (See Figure 4C).

### Morphological effect of Revacept on ischemic cerebral stroke by MCA occlusion

**Figure 5:**
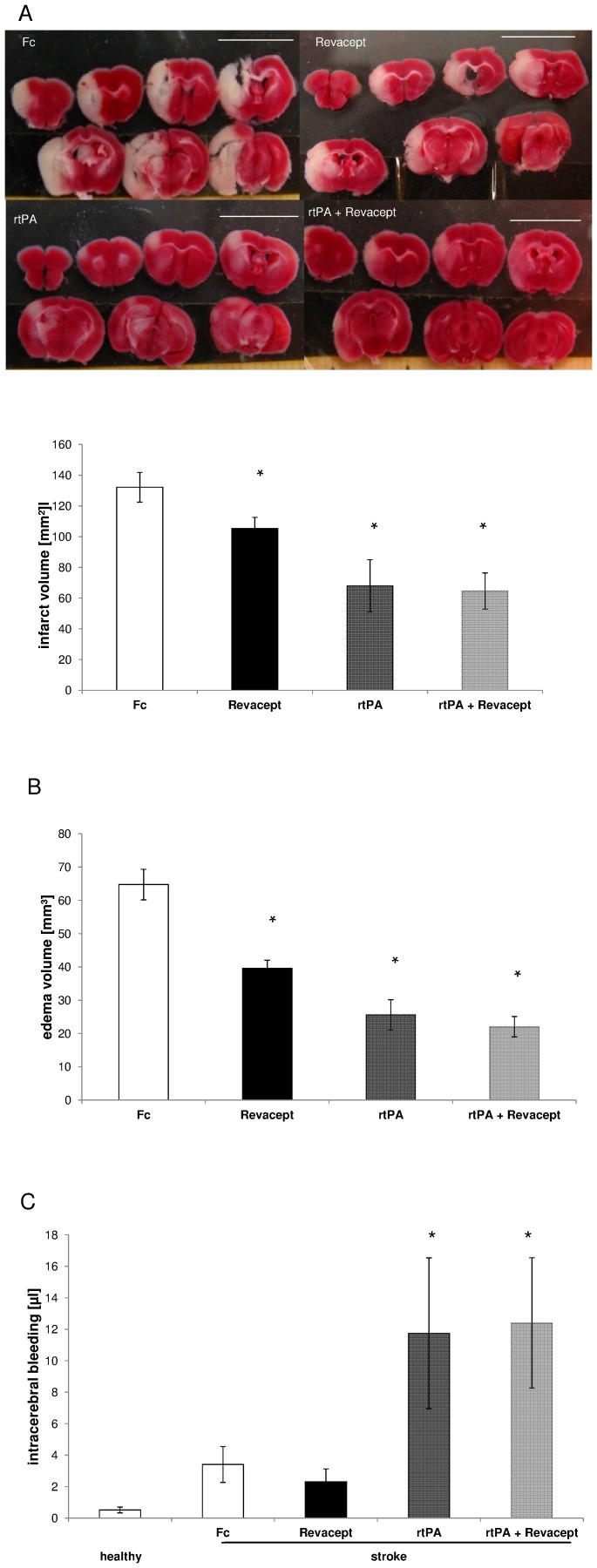
Assessment of stroke volume by post-mortem 2,3,5-TTC staining in brain slices from mice 24 hours after reperfusion following a 1 hour transient MCA occlusion. (A) representative 2,3,5-TTC stained brain slices. The white scale bar corresponds to 1 cm (upper panel). Mean infarct volumes (lower panel). (B) Mean edema volumes. (C) mean bleeding volumes, as assessed by measuring cyano-methemoglobin content of tissue extracts. Means ± SEM from n = 9 animals per group are given, * denotes significant differences of p<0.05 compared to Fc. At the beginning of reperfusion, mice received either Revacept (1 mg/kg) or an equimolar amount of Fc only (0.33 mg/kg), or rtPA (10 mg/kg), or a combination of Revacept and rtPA, intravenously.

24 hours after MCA occlusion, the cerebral infarct volume of mice was significantly reduced after treatment with Revacept, as well as with rtPA, and with the combination of both drugs (see Figure 5A). Revacept, however, was significantly less effective compared to rtPA or the combination therapy. Additionally, the edema volume was significantly reduced after Revacept, as well as with rtPA treatment, and with the combination of both, in these mice with ischemic stroke (see Figure 5B). Again, Revacept was less effective in reducing cerebral edema compared to rtPA or combination therapy.

Upon measuring cyano-methemoglobin content in minced mouse brain sections, no signs of increased intracranial haemorrhage after Revacept treatment occurred, as assessed 24 h and 72 hours after stroke. 24 h after stroke, there was no difference between Revacept and the Fc only control group undergoing MCA occlusion and stroke (please see Figure 5C). In contrast, rtPA led to an increase in intracerebral bleeding volume compared to Fc only and to Revacept. No additional bleeding occurred in the group treated with Revacept in addition to rtPA compared to treatment with rtPA alone (Figure 5C). In healthy mice which had not been subject to any intervention, a minimal background signal is most likely caused by remaining intravascular haemoglobin.

Also 72 hours after stroke, bleeding volume was increased to 0.931±0.32 µl in mice treated with rtPA, whereas it amounted to only 0.32±0.09 and to 0.25±0.1 µl in mice treated with Revacept or Fc only, respectively (n = 4 animals in each group, p<0.05 rtPA versus either Revacept or Fc).

### Effect of Revacept on cellular inflammatory infiltration and reperfusion damage after ischemic cerebral stroke by MCA occlusion

**Figure 6:**
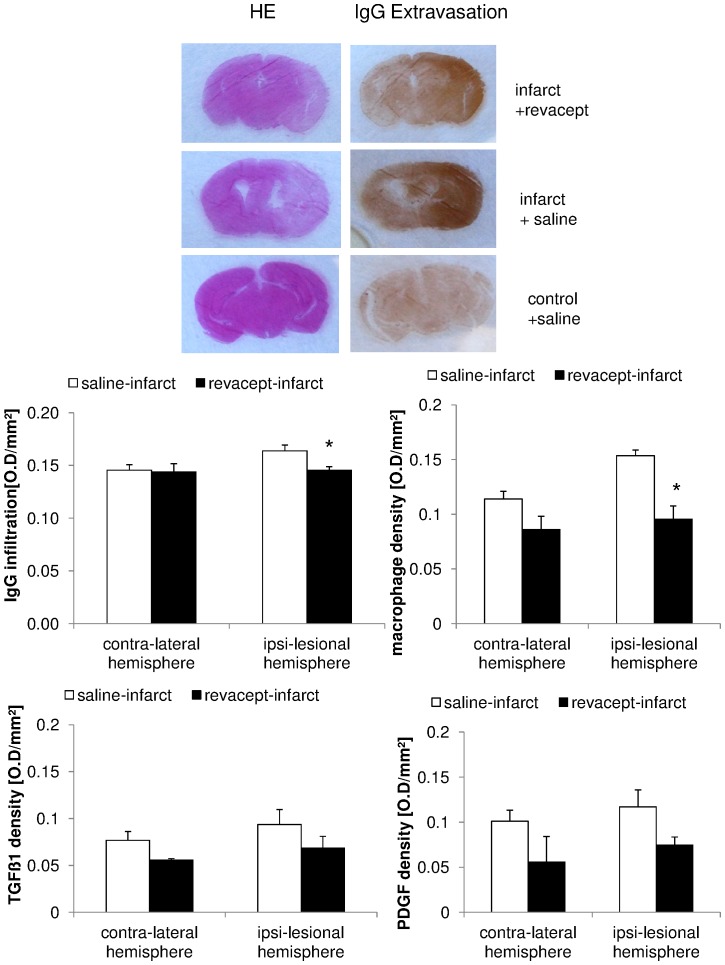
Detection of parameters of cellular activation and inflammation in brain tissue by in situ immune-histochemistry in mice. Top panels show representative brains section of mice with standard hematoxillin eosin staining (HE), and specific IgG extravasation after MCA occlusion and stroke. Brains from Revacept- and saline- treated stroke mice and non-ischemic control brain areas are representatively demonstrated. Bar graphs show immuno-fluorescence signals for immunoglobulin G (IgG), macrophages, transforming growth factor beta (TGF beta) and platelet derived growth factor (PDGF) in mice with stroke after MCA occlusion. We compared the effect of Revacept (1 mg/kg) or Fc only (0.33 mg/kg)-treated mice in the infracted left hemisphere and the right hemisphere in the absence of ischemia. The mean integrals of immuno-fluorescence signals in the respective group are shown as arbitrary units ± SEM, n = 3 per group * indicate significant differences of p<0.05 to saline treated mice.

24 hours after MCA occlusion, several parameters which indicate inflammatory response to injury were assessed by in situ immuno-histochemistry in brain sections of mice. There was a significantly reduced detection of immunoglobulin G (IgG) after treatment with Revacept, as compared to Fc only (see Figure 6) and reduced density of macrophages with Revacept. Additionally, there was a trend towards reduction of TGFß and PDFG expression after Revacept in mice with ischemic stroke (see Figure 6).

## Discussion

In this study, we demonstrate that treatment with Revacept (recombinant dimeric GPVI-Fc) leads to a significant reduction of thrombus formation after endothelial damage as well as a significant reduction of cerebral infarction and improvement of functional and prognostic outcome in ischemic stroke. This anti-ischemic effect is achieved without increasing risk of intra-cerebral hemorrhage. Thus, Revacept (soluble GPVI-Fc) efficiently improves the outcome of ischemic stroke without increasing the bleeding risk. This effect is due to the capacity of Revacept to competitively inhibit the binding of platelet GPVI and of soluble von Willebrand factor (vWF) to collagen, thereby blocking collagen-mediated, but also vWF-mediated platelet activation. The risk-benefit ratio of Revacept was superior to that of rtPA alone, or to the combination of both, which showed efficacy, but increased risk of bleeding.

**Figure 7:**
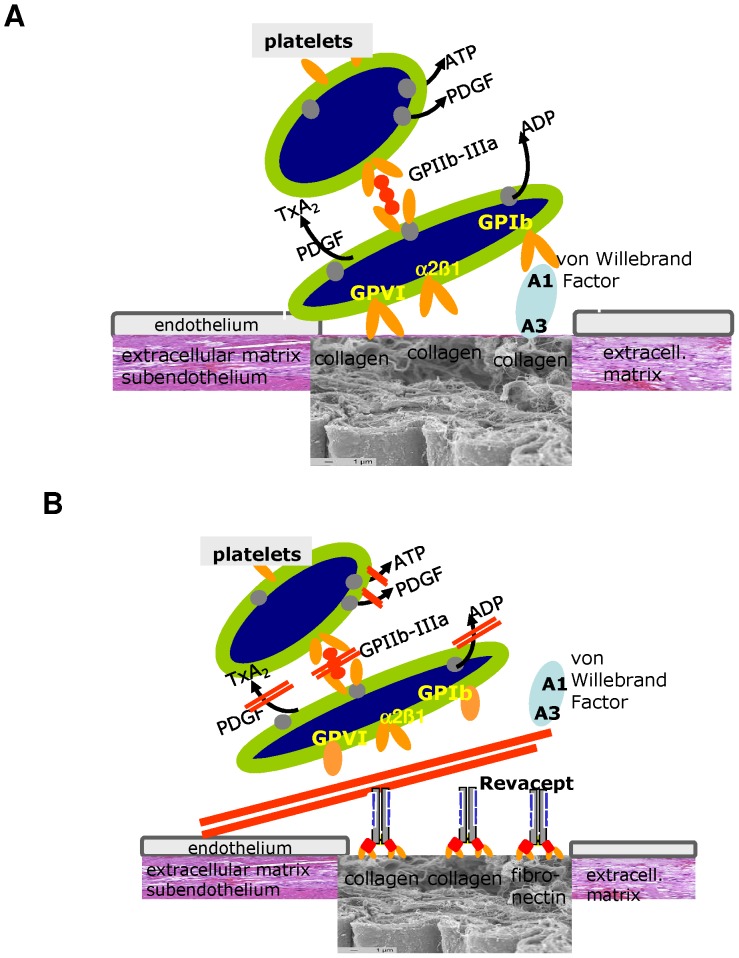
Structure and mode of action of Revacept. (A) Simplified model of platelet adhesion, aggregation and activation and arterial thrombosis at site of vascular injury. Specific interaction of the extracellular matrix with the platelet receptors alpha_2_ beta_1_; glycoprotein VI (GPVI) and glycoprotein Ib (GPIb). (B) Revacept binds to collagen exposed at vascular injury. Revacept prevents the first steps of von Willebrand Factor (vWF) and collagen-mediated platelet activation and platelet aggregation. Thus, Revacept seems a useful tool for the treatment or prevention of stroke, in which both vWF and collagen-mediated platelet activation play a central role. For reason of clarity, other pathways that are also relevant to platelet activation in stroke have been omitted.

vWF and collagen-mediated platelet activation play a central role in the development and propagation of ischemic cerebral stroke (please see Figure 7A).

The initial platelet activation via the platelet receptors GPIb by collagen-bound vWF or GPVI by collagen, leads to activation of the integrin αIIbβ3 (GPIIb/IIIa) platelet receptor, and to the consecutive steps of platelet aggregation and spreading, and to pathological thrombus formation (for review see references [4], [16], [17]). For this reason, the GPIIb/IIIa receptor was also investigated as a target for anti-platelet therapy in the same stroke model in mice [8], [9]. However, anti-GPIIb/IIIa antibodies were ineffective in reducing the infarct size, and animals developed intracerebral hemorrhage with more than 50% lethality. Additionally, tail bleeding time was markedly prolonged, even much more than with anti-GPIb antibodies [8]. Abciximab, a GPIIb/IIIa-inhibitor, which has been successfully used for the treatment of acute coronary syndrome, was also investigated for the treatment of acute ischemic stroke in the Abciximab emergent stroke treatment trial (AbESTT) [18]. Despite favourable results in this initial phase II study with only mild bleeding complications, a clinical phase III trial (AbESTT-II) was discontinued due to increased fatal intracranial haemorrhage and poor outcomes [10].

Inhibition of the GPVI pathway is feasible by administration of the anti-GPVI antibody JAQ1, which led to a depletion of the GPVI protein but also to an inhibition of other platelet signalling pathways, such as thrombin-dependent activation [19], [20] Another tool to interfere with the GPVI-mediated platelet activation is the soluble GPVI fusion protein GPVI-Fc (Revacept)(see Figure 7B). Administration of Revacept led to a reduction in platelet adhesion to the injured vessel wall in healthy mice [21] as well as in cholesterol-fed ApoE -/- mice [14] and reduced neointima formation [22]. The results of the present study (see Figure 2) confirm the inhibitory effect of Revacept on platelet-collagen interaction in an arterial wall injury model. In this previous study [21], dose-finding was investigated. The dose-range of Revacept was further confirmed in this study with experimental deep vascular injury (Figure 2). This effect is observed with Revacept, but not with some other GPVI-Fc fusion proteins [23], which might be due to detail differences in the protein structure especially the linker between the GPVI domain and Fc part. Revacept also led to an improvement in motor function, reduction in infarct volume and edema in ischemic stroke, without an increased risk of intra-cerebral haemorrhage. Even if this is not a head to head comparison, compared to the previous findings of Kleinschnitz et al [8], the inhibitory effect on functional outcome of Revacept was more pronounced compared to treatment with anti-GPVI antibodies, despite the methodological difference that the anti-GPVI-antibodies were given before the onset of occlusion. In contrast, Revacept was given after 1 hour of arterial occlusion during reperfusion, since this approach rather corresponds to the situation in which patients with stroke present in the clinic.

One significant difference in the mode of action to explain this marked increase in efficiency is that Revacept is also able to inhibit binding of vWF to collagen (Figure 1C). Since GPVI and vWF have different binding sites on collagen [24], [25] we assume that Revacept inhibits vWF binding via the A3 binding domain to collagen via steric hindrance leading to a potentiating effect in the inhibition of platelet adhesion and a higher efficacy than the anti-GPVI-antibody (see Figure 7 B for illustration). This cross – inhibition to collagen binding was also described in a previous study with another collagen-binding protein, LAIR-2-Fc. This soluble collagen receptor, physiologically found on immune cells, binds to the collagen epitope for GPVI (GPOx) and also inhibited the binding of vWF to collagen [26]. The same inhibition of vWF binding to collagen could also be shown in the present study with Revacept, which is known to also recognize the GPO repeat in collagen.

VWF is also described as a pro-inflammatory mediator of leukocyte extravasation [27] which is - among others - one important step during the onset of ischemia leading to pro-inflammatory activity in the ischemic brain (for review see reference [28]). Thus, Revacept might also inhibit extra-vasation of inflammatory cells and reduce intra-cerebral inflammatory activity which also affects function of the blood brain barrier, finally leading to a reduction of edema area. Besides the effect on cerebral infarction and edema, we found evidence for increased white blood cell infiltration measured by immuno-histochemical analyses of IgG retention, which is markedly increased in ischemic brain tissue.This effect is slightly ameliorated by Revacept (Figure 6). Similarly, infiltration of macrophages in the infarcted brain region was reduced by therapy with Revacept (Figure 6). Thus Revacept might have anti-inflammatory properties in addition to its anti-platelet effects by blocking VWF binding to collagen in injured vessels. According to our results, the experience from the AbESTT trial and elegant work from Meyer et al, the interaction of VWF with the vascular collagen seems to play a pivotal role in cerebral stroke [29]. These authors excluded a significant role of VWF interaction with GPIIb/IIIa. However, the situation remains complex as others [30] found a marked reduction of VWF –mediated platelet adhesion and marked reduction of cerebral infarct volume in a similar mouse stroke model with GPIIb knockout. Thus it seems plausible that Revacept might inhibit the initial VWF binding to injured or activated vasculature during ischemia and reperfusion, and by preventing the above mentioned complex consecutive steps exerts beneficial effects during cerebral ischemia.

In this study, we found improved reperfusion after MCA occlusion by the combination therapy of Revacept and rtPA, as measured by transcranial Doppler (Figure 3). The reperfusion was comparable to placebo after therapy with either agent alone. Therefore, we conclude that Revacept may interrupt the mechanisms which are responsible for loss of the primary microvessel permeability barrier which frequently impacts on reperfusion after cerebral ischemia leading to a “no-reflow phenomenon” as previously described for other anti-platelet interventions [30]. Interestingly, the strongest reduction in infarct area was observed in the most caudal 2 brain sections where blood supply during middle cerebral artery occlusion might be partially preserved from collaterals from the caudal cerebral artery, originating from the basilar artery, assuming that Revacept is especially effective in reducing the ischemic penumbra after MCA occlusion. The increased reperfusion with combined rtPA and Revacept treatment is most likely due to the thrombolytic effect and anti-platelet effect. Whether this results in increased thrombolytic efficacy, the prevention of novel thrombo-embolism or local effects in the reperfusion which ameliorate the no-reflow phenomenon is a general debate about the effects of platelet inhibitors. Certainly it remains to be clarified for the effects of Revacept (please also compare for review [31]).

The acute survival of these mice after experimental stroke and Revacept treatment was somewhat worse over the first 48 hours than the clinically established treatment with rtPA(Figure 4C). Intracranial bleeding volume, however, was larger in rtPA-treated than in Revacept-treated mice (Figure 5C). In order to confirm that the effect on the prognosis and on safety was not a transitional phenomenon, we extended the observation period to 72 hours. After 72 hours, Revacept alone and the combination with rtPA was comparable to rtPA. Most importantly, the absence of any additional intracranial bleeding with Revacept was also found in the mice after an observation period of 72 hours.

Compared to previously published results with filament occlusions of the middle cerebral artery, rtPA had a strong beneficial effect on infarction volume and functional outcome. Some investigators [32]; [33] found a paradoxical increase of the infarction volume in rtPA treated mice after middle cerebral artery occlusion, whereas others [34], [35] with different ischemia models could not confirm these findings. Thus a large controversial debate on these findings is going on, which is most likely due to small methodological differences in the treatment of these mice and even more likely due to differences in anesthesia. Recently it was reported that the effect of rtPA was dramatically different between awake and anesthesized mice and that e.g. ketamine plus rtPA has largely reduced the cerebral infarct volume in MCAO in mice [36].

In a previous phase I study in humans, Revacept proved safe with regard to bleeding time, general coagulation and platelet counts in healthy volunteers [13]. Thus, the lesion-specific binding of Revacept to collagen at the injured vessel wall does not influence the general platelet function including its receptors, and presumably does not incur a risk of bleeding complications or intra-cerebral haemorrhages during the treatment of stroke. However no impact on general platelet activation was found, since ADP- and thrombin-induced platelet activation was unaffected [13]. This finding is in contrast to the broad-range inhibition seen with anti-GPVI antibodies in animal models [19]. On the other hand, Revacept is able to specifically inhibit functional platelet activation, and leads to a clear inhibition of thromboxane release from human platelets (Figure 1D).

In conclusion, we assume that Revacept blocks the GPVI-mediated and also the vWF-mediated platelet activation and thrombus formation by masking vascular collagen after vascular injury. This could also happen in the microcirculation of the injured ischemic tissue during ischemia and reperfusion without major vascular damage in accordance with a previous study [29]. Thus Revacept – other than existing anti-platelet drugs - inhibits lesion-specifically the initial steps of platelet adhesion and aggregation during complications of atherosclerotic disease, such as acute ischemic stroke, without affecting the function of circulating platelets or other functions of the haemostatic system. Revacept might therefore be a promising, effective and safe drug for the treatment of ischemic complications in stroke and reperfusion e.g. by improving the thrombolytic efficacy of rtPA, or during intravascular cerebral interventions, such as thrombectomy, e.g by ameliorating the no reflow phenomenon after successful recanalisation [37].

Further studies are required to elucidate the different underlying mechanisms of the beneficial role of Revacept during ischemia and reperfusion especially to clarify the mechanism of the anti-inflammatory effects.

## Supporting Information

Figure S1 **Histological investigation of the endothelial lesion in the carotid artery of a mouse.** The endothelial layer is destroyed after vigorous ligation of the common carotid artery and the subendothelium is exposed. In contrast, endothelial cells are visible in native carotid artery (see arrows, Hematoxillin Eosin Staining). DCF labelled platelets attach to the vascular lesion (fluorescent image).
(PPT)

Figure S2 **Representative images of platelet aggregation in vivo.** As assessed in the carotid artery by using intravital microscopy (IVM). Thrombus formation after vascular injury induced in the left common carotid artery. Administration of Revacept (1 mg/kg) was compared to an equimolar amount of Fc only (0.33 mg/kg). The agents were injected intravenously (tail vein) before experimental endothelial lesion by thread ligature. To visualize platelet adhesion and thrombus formation, mice received DCF fluorescence-labelled platelets before injection of agents.
(PPT)

Methods S1 Materials and Methods
(DOC)
