# Supplementary material for: The GPVI-Fc Fusion Protein Revacept Improves Cerebral Infarct Volume and Functional Outcome in Stroke
Source: PLoS One. 2013 Jul 23;8(7):e66960. doi: 10.1371/journal.pone.0066960 (PMC3720811; doi:10.1371/journal.pone.0066960)
Supplement: Figure S1 — Histological investigation of the endothelial lesion in the carotid artery of a mouse. The endothelial layer is destroyed after vigorous ligation of the common carotid artery and the subendothelium is exposed. In contrast, endothelial cells are visible in native carotid artery (see arrows, Hematoxillin Eosin Staining). DCF labelled platelets attach to the vascular lesion (fluorescent image). (PPT) [file pone.0066960.s001.ppt]

## Slide 1
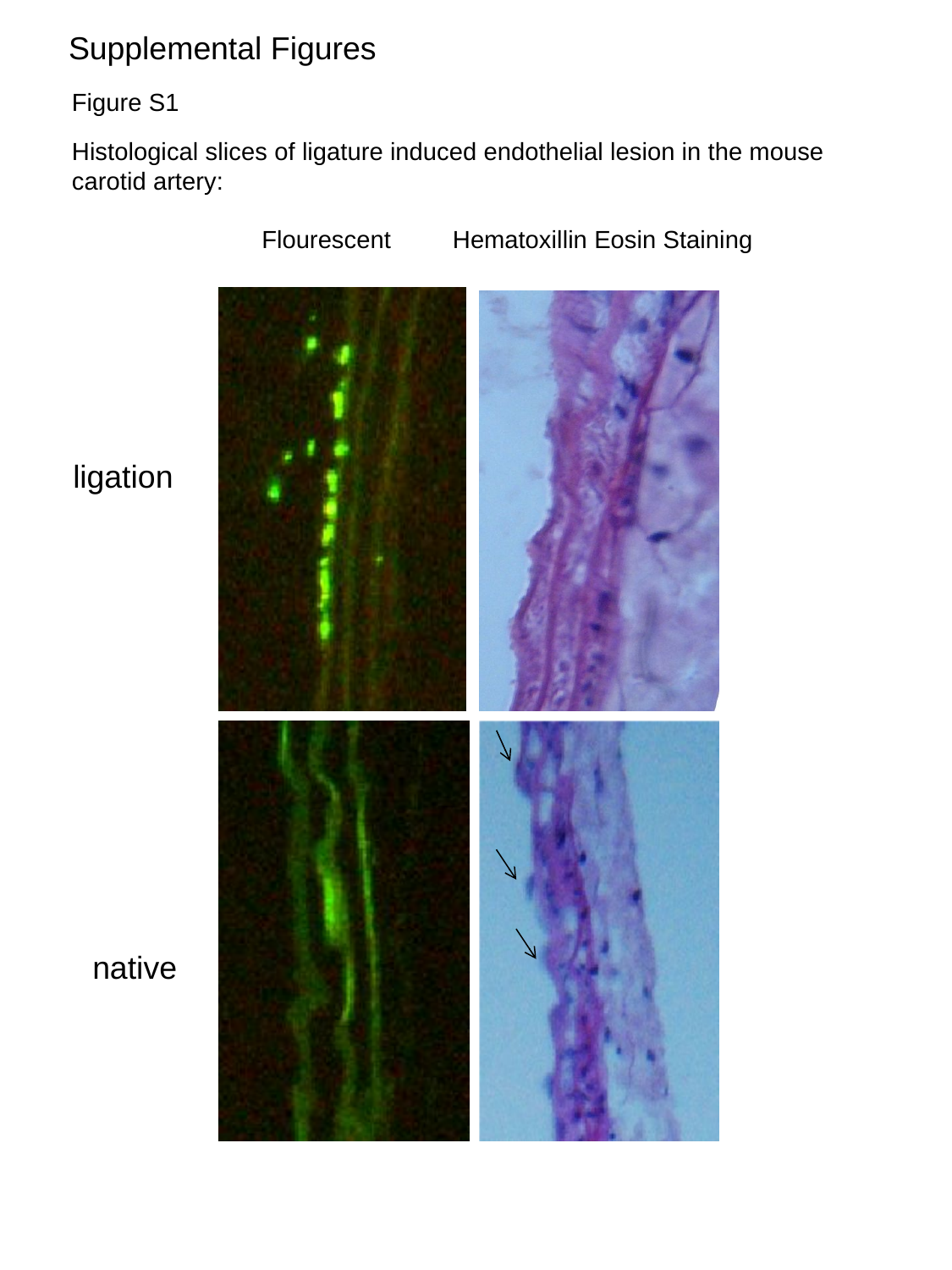

Supplemental Figures
Figure S1
Histological slices of ligature induced endothelial lesion in the mouse carotid artery:
	 Flourescent 	Hematoxillin Eosin Staining
ligation
native
