# Supplementary figures and images for: The GPVI-Fc Fusion Protein Revacept Improves Cerebral Infarct Volume and Functional Outcome in Stroke
Source: PLoS One. 2013 Jul 23;8(7):e66960. doi: 10.1371/journal.pone.0066960 (PMC3720811; doi:10.1371/journal.pone.0066960)

## Slide 1
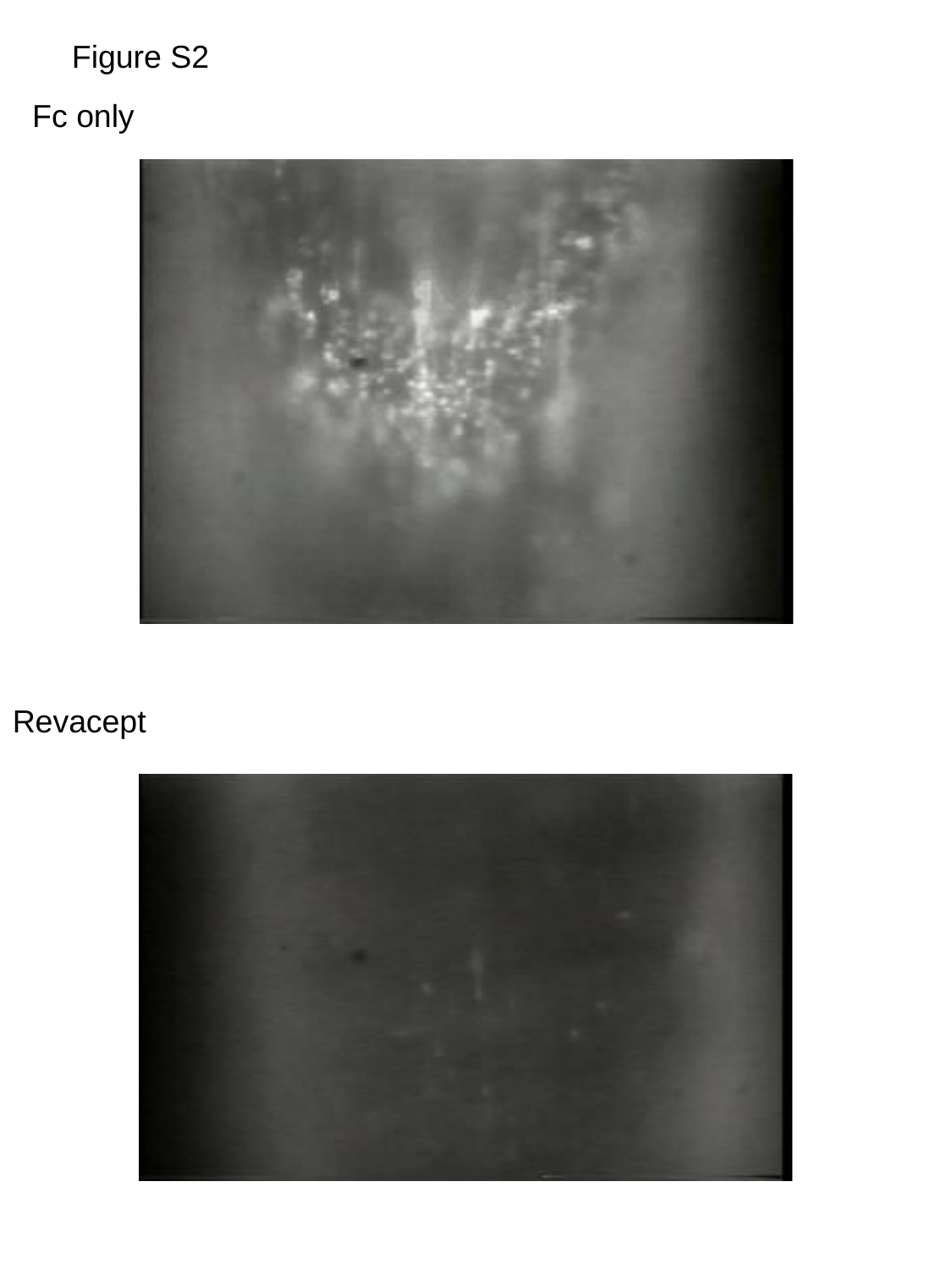

Figure S2
Fc only
Revacept

Supplement: Figure S2 — Representative images of platelet aggregation in vivo. As assessed in the carotid artery by using intravital microscopy (IVM). Thrombus formation after vascular injury induced in the left common carotid artery. Administration of Revacept (1 mg/kg) was compared to an equimolar amount of Fc only (0.33 mg/kg). The agents were injected intravenously (tail vein) before experimental endothelial lesion by thread ligature. To visualize platelet adhesion and thrombus formation, mice received DCF fluorescence-labelled platelets before injection of agents. (PPT) [file pone.0066960.s002.ppt]
