# Supplementary material for: The GPVI-Fc Fusion Protein Revacept Improves Cerebral Infarct Volume and Functional Outcome in Stroke
Source: PLoS One. 2013 Jul 23;8(7):e66960. doi: 10.1371/journal.pone.0066960 (PMC3720811; doi:10.1371/journal.pone.0066960)
Supplement: Methods S1 — Materials and Methods (DOC) [file pone.0066960.s003.doc]

Material & Methods S1

Experimental groups and materials used

As therapeutic tool we used Revacept, a dimeric soluble GPVI-Fc which was produced as previously described ^1^ as well as the Fc part from human IgG for the control group ^2^. In various interventions, recombinant tissue plasminogen activator (rtPA, Actilyse, Boehringer Mannheim, Germany), and rtPA in combination with Revacept was used.

**ELISA to determine binding and competition of Revacept to collagen**

Demonstration of Revacept-specific collagen binding was performed with an immuno absorbent ELISA assay.  A micro-titer plate was coated with 10 µg/ml type I mouse or bovine collagen (BD Bioscience) in sodium carbonate coating buffer.  After blocking with Roti-block (Roth, Germany), samples and standards were added onto micro titer plates. After washing with PBST, a horse radish peroxidase-coupled goat anti-human IgG antibody (27 ng/ml) was added resulting in the formation of a sandwich complex of solid phase collagen-Revacept with the enzyme-labelled antibody. The micro-titer plate was washed to remove unbound reactants and TMB substrate was used for detection. After stopping the reaction with 1 M H_2_SO_4_, the amount of hydrolysed substrate was read using a micro-plate reader at 450 nm (reference wavelength 690 nm) and was directly proportional to the concentration of bound Revacept on the immobilized collagen.

**ELISA to determine binding and competition of vWF to collagen**

An ELISA was established to evaluate binding of human vWF to mouse or bovine collagen I. A 96 well MaxiSorp plate (NUNC) was coated with 10 µg/ml mouse or bovine collagen I o/n (BD Bioscience) in carbonate coating buffer. Wells were blocked with 1x Roti-Block (Roth). After rinsing, they were incubated with PBS (Biochrom AG), 0.1% Tween-20 for 100 minutes with increasing dilutions of human vWF (Abcam Inc.) at room temperature. After washing, wells were incubated with a polyclonal rabbit anti-human vWF antibody with horse radish peroxidase (HRP) conjugate in a dilution of 1: 3000 or 1:5000 in PBS, 0.1% Tween-20 for 75 minutes at room temperature. After rinsing, 1-Step Ultra TMB-ELISA substrate was added and incubated until blue staining developed. Reactions were terminated by addition of sulfuric acid and extinctions were determined at 450 and 595 nm with a Tecan Infinite F200 ELISA reader. In competition experiments 300 ng/ml of human vWF factor (which was in the linear binding range to collagen and yielded an OD measurement of about 1) was incubated on collagen type I-coated plates with increasing dilutions of Revacept ranging from 10 – 10.000 nmol/L or equimolar amounts of Fc only.

**ELISA to determine the release of thromboxane from human platelets in response to stimulation with collagen-related peptide (CRP)**

Human platelet-rich plasma (PRP) was obtained from volunteers by cautious blood sampling. To measure the influence of Revacept on aggregation, various amounts of Revacept (3.13, 6.25, 12.5 or 25 µg/ml) or buffer were spiked into PRP containing endogenous vWF. Aggregation of PRP was induced by addition of 1 µg/ml CRP-XL (cross-linked collagen-related peptide). After incubation at room temperature for 30 minutes, samples were diluted to 1:100 or 1:10 (untreated control) with assay buffer. Release of thromboxane B2 to the supernatant was analyzed using a commercial ELISA (Abnova, Taiwan). Detection of the B2 subtype has been shown to closely correspond to release of thromboxane A2, and is a more stable metabolite allowing for more reliable measurement by ELISA.

Animal studies

Experiments were approved by the local animal welfare authority in Munich, Germany (Regierung von Oberbayern, Sachgebiet Tierschutz, reference number 55.2-1-54-2531-98-09). 8-12 week old male C57Bl/6J mice weighing 21 to 29g were used (Charles River, Sulzfeld, Germany) and housed under standard conditions with a 12 hour diurnal cycle and free access to food and water. To test the antithrombotic effect of Revacept on an injured arterial wall, a lesion of the endothelium was induced by transient ligature of the left common carotid artery (ACC) as previously described ^3^. To visualize platelet adhesion to the injured vessel wall under in vivo conditions, platelets were fluorescently labelled with 5-(and-6)-carboxyfluorescein diacetate, succinimidyl ester (5(6)-CFDA, SE (DCF, Invitrogen), injected intravenously and monitored in situ with an intravital microscope (IVM) over 60 minutes after the endothelial damage as described before ^3^. Administration of Revacept or Fc only, respectively, was performed immediately before inducing the endothelial lesion in the ACC.

The influence of Revacept on arterial thrombosis induced by deeper lesions of the arterial wall was investigated in a mouse model of wire-induced vascular injury. After preparation of the carotid artery, a coronary guiding wire was introduced via the external carotid artery and rubbed over the endothelium of the mouse common carotid artery (C57BL/6J mice). Revacept or control was injected intravenously before the intervention. 4 hours after the intervention animals were infused with 20 ml of saline solution through their tail veins, followed by a second infusion of 4 mL paraformaline (PFA, 4%). Then, the large vessels including both carotid arteries were dissected free, opened longitudinally and spread out on microscopic cover slips. Thrombus size was quantified after digital imaging and quantification by image analysis software (Photoshop, Adobe).

The effect of Revacept on cerebral infarction and neurological function / motor activity after cerebral ischemia was assessed in mice after occlusion of the left middle cerebral artery (MCA). One hour ischemia was induced by placing a silicon-coated monofilament (Doccol, USA) in the MCA via the left common / internal carotid artery as described by Hata, Hermann and co-workers ^4, 5^. Flow reduction in the MCA was monitored with a laser Doppler flow probe attached to the left temporal skull. At the beginning of reperfusion, Revacept or Fc only, respectively, were injected via the tail vein. rtPA (10 mg/kg bodyweight) in combination with Heparin (200 IU / kg bodyweight) was also studied in this stroke model. After reperfusion times of 4 hours, 24 hours, or 72 hours mice underwent evaluation for neurological and motor dysfunction. Survival after stroke was monitored for 4, 24 and 72 hours after reperfusion. 24 or 72 hours after reperfusion of the middle cerebral artery, mice were sacrificed, the brain was removed and the infarct area investigated by 2,3,5-triphenyltetrazolium (TTC) staining. Additionally, brains were investigated for intracerebral hemorrhage by a spectrophotometric assay.

During all interventions, anaesthesia was induced with Medetomidin 0.5 mg/kg (Domitor®, Janssen-Cilag GmbH), Midazolam 5 mg/kg (Dormicum®, Roche) and Fentanyl 0.05 mg/kg (Fentanyl®-Janssen, Janssen-Cilag GmbH) and maintained with Isoflurane 0.2 – 0.8% (Isofluran CP®, CP-Pharma), Analgesia was achieved with 200 mg/kg Metamizol p.o. (Novalgin®, Sanofi-Aventis) three times within 24 hours.

**Determination of platelet function *in vivo* (intravital microscopy)**

An intra vital microscope (Axioscop2 FS mot, Carl Zeiss) equipped with a video camera (BC-71 b/w-CCD-Camera, Horn Imaging), a DVD recorder (RDR-AT205, Sony) and a video screen (X-15A, AG neovo technologies) was used. For detecting the vessel diameter, a video was performed at 100-fold magnification. For determination of transiently adherent platelets, video sequences of 30 seconds were recorded at 200-fold magnification 5, 10, 15, 20 and 30 minutes after endothelial damage. Transiently adherent platelets were counted in slow motion during 30 second video sequences within a 150 x 100 µm² window which was placed on the video screen directly over the endothelial lesion. 30 and 60 minutes after endothelial damage the platelet thrombus area was determined. For the determination of the thrombus area, 3 screen shots were taken and the area of mean total platelet thrombi were added up to an overall thrombus area and evaluated with an image analysis program (Photoshop® CS5, Adobe).

Morphological and functional outcome after ischemic stroke

Assessment of neurological function and motor function was performed 4, 24 and 72 hours after reperfusion of the MCA. The motor function was evaluated with a grip strength test (Bio-GS3, Bioseb, France) in 5 consecutive measurements. The mean value of these measurements was determined and the percentage change compared to the value before surgery was determined. The neurological function was assessed with a modified Bederson-score ^6^: no spontaneous movement was scored as 4 points, circling as 3, decreased resistance to lateral push without circling as 2, forelimb flexion to one site at tail lifting as 1 and no deficit as 0. Additionally, survival was monitored at 4, 24 and 72 hours after reperfusion of the MCA.

Assessment of brain morphology was performed in mice 24 after middle cerebral arterial occlusion. After lethal anaesthesia of the mice, brains were quickly removed and seven 1-mm-thick coronal sections were cut starting from the frontal pole using a Leica Microtom CM 1850 cryostat with a mouse brain slice matrix (Cat BSM001.1, Zivic Lab Inc) (4°C). Infarct area was visualized by staining with 2% 2,3,5-triphenyltetrazolium chloride (TTC; Sigma Aldrich No 93140,) buffered in PBS (Biochrom AG) with pH 7.6 – 7.4 at room temperature for 30 minutes. Brain slices were digitally photographed and the infarct size was quantified by image analysis software (Photoshop® CS5, Adobe) by researchers blinded to the treatment groups.

The hemoglobin content of brains was quantified with a spectrophotometric assay 24 and 72 hours after MCA occlusion. In brief, frozen brain tissue was homogenized on dry ice and consecutively dissolved with distilled water. After centrifugation the hemoglobin-containing supernatant was collected, 80 µL of Drabkin's reagent (Sigma) was added to a 20 µL aliquot. Cyanomethemoglobin with an absorbance peak at 540 nm was determined by measuring the OD of the solution at ≈550 nm wavelength. The absorption of mouse brains was compared to mouse blood incubated with brain tissue to generate a standard absorbance curve for quantification.

**Immunohistochemistry**

Both, healthy mice and animals subjected to MCA occlusion were terminally anesthetized. Brains were removed, washed in ice-cold PBS, and embedded in TissueTek freezing medium (Sakura Finetek, the Netherlands), frozen on dry ice and stored at −80°C. 10 µm cryostate coronal sections were processed for avidin-biotin-peroxidase immunostaining using a Leica Microtom CM 1850 cryostat. After postfixation in 4% PFA pH 7.4 and acetone, endogenous peroxidase activity was quenched in 0.6% H_2_O_2_/methanol, unspecific binding was blocked with 20% rabbit serum (DAKO, Hamburg, Germany) in PBS. Goat anti-mouse IgG antibody (Santa Cruz Biotechnology), goat anti-mouse TGFß1 antibody (Santa Cruz Biotechnology) and goat anti-mouse monocloncal antibody against PDGF-C (Santa Cruz Biotechnology), or rat anti-mouse F4/80 monocloncal antibody was used for macrophage detection (BMA biomedicals) and applied over night at 4°C.

The sections were washed with PBS, incubated at RT with biotinylated secondary mouse anti-rat or mouse anti-goat biotinylated antibody (DAKO, Hamburg, Germany, 1:300) and followed by incubation with streptavidin-conjugated HRP for 30 min, (DAKO, Hamburg, Germany, 1:100) and developed in peroxidase substrate solution (Liquid DAB+ Substrate Chromogen System, DAKO, Hamburg, Germany). After counterstaining with Harris’ Hematoxylin, sections were dehydrated and mounted with Permount (Fisher Scientific, Schwerte, Germany). Sections were viewed under a Zeiss Axiovert Microscope (20 x magnification), digitally photographed. Positive immunostaining was quantified separately in the right and left hemisphere using 150 µl of TMB solution (Thermo) mounted onto the slices. After 4 minutes the colouring dye was removed and transferred on microtiter plates into a Tecan Sunrise ELISA reader for automated quantification of staining signal. All quantifications were performed on three sections per mouse spaced 2 mm apart and average values from three optical densities per square micrometer were determined for each animal.

**References**

1. Ungerer M, Rosport K, Bültmann A, Piechatzek R, Uhland K, Schlieper P et al. Novel Antiplatelet Drug Revacept (Dimeric Glycoprotein VI-Fc) Specifically and Efficiently Inhibited Collagen-Induced Platelet Aggregation Without Affecting General Hemostasis in Humans. *Circulation.* 2011;123:1891-1899.

2. Zeibig S, Li Z, Wagner S, Holthoff HP, Ungerer M, Bültmann A et al. Effect of the oxLDL binding protein Fc-CD68 on plaque extension and vulnerability in atherosclerosis. *Circ Res.* 2011;108:695-703.

3. Massberg S, Gawaz M, Grüner S, Schulte V, Konrad I, Zohlnhofer D et al. A crucial role of glycoprotein VI for platelet recruitment to the injured arterial wall in vivo. *J Exp Med.* 2003;197:41-49.

4. Hata R, Maeda K, Hermann D, Mies G, Hossmann KA. Evolution of brain infarction after transient focal cerebral ischemia in mice. *J Cereb Blood Flow Metab.* 2000;20:937-946.

5. Hermann DM, Kilic E, Hata R, Hossmann KA, Mies G. Relationship between metabolic dysfunctions, gene responses and delayed cell death after mild focal cerebral ischemia in mice. *Neuroscience.* 2001;104:947-955.

6. Bederson JB, Pitts LH, Tsuji M, Nishimura MC, Davis RL, Bartkowski H. Rat middle cerebral artery occlusion: evaluation of the model and development of a neurologic examination. *Stroke.* 1986;17:472-476.
